# Cardiotoxicity of Immune Checkpoint Inhibitors and Chimeric Antigen Receptor T-Cell Therapy

**DOI:** 10.3390/jcdd13080385

**Published:** 2026-08-12

**Authors:** Ahmad Naser, Ahmed Ashraf Morgan, Lili Zhang

**Affiliations:** 1 Department of Internal Medicine, Jacobi Medical Center, Albert Einstein College of Medicine, Bronx, NY 10461, USA; 2 Division of Cardiology, Department of Medicine, Montefiore Medical Center, Albert Einstein College of Medicine, Bronx, NY 10467, USA

**Keywords:** immune checkpoint inhibitors, cardiotoxicity, immune checkpoint inhibitor myocarditis, CAR T-cell cardiotoxicity, cardiomyopathy, accelerated atherosclerotic cardiovascular disease

## Abstract

Over the last decade, immunotherapy has transformed oncology, with an ever-expanding list of indications. As with any therapeutic class, these agents are accompanied by adverse events that are increasingly recognized and pose new challenges for patient care expanding the scope of cardio-oncology. Using the PubMed database, this narrative review summarizes the cardiovascular adverse events associated with the most commonly used immunotherapies, immune checkpoint inhibitors (ICIs), with a focus on the mechanisms, epidemiology, diagnosis, treatment, and monitoring of ICI-associated myocarditis. We also review accelerated atherosclerotic disease, heart failure, cardiomyopathy (including stress-induced cardiomyopathy), arrhythmias, and pericardial disease related to ICI therapy. Finally, we outline CAR T-cell therapy associated cardiotoxicity, including cytokine release syndrome.

## 1. Introduction

Cancer immunotherapy is a rapidly growing field that utilizes different classes of medications that share the same goal of utilizing the immune system in fighting cancer through different mechanisms. Currently, the most commonly used of them are immune checkpoint inhibitors (ICI) and chimeric antigen receptor (CAR) T-cells. Other emerging therapies include cytokines, chemokines, cell therapies (including genetically engineered cells), checkpoint modulatory antibodies, cancer vaccines, bispecific T-cell engagers (BiTEs), dual-specific antibodies, small molecules, oncolytic viruses, and immune adjuvants [1]. For the purposes of this review, the discussion will focus mainly on ICI and CAR T-cell therapy.

A comprehensive literature search was performed using the PubMed database to identify relevant studies published from database inception through 2025. Search terms were selected to capture the breadth of research related to the topic, and Boolean operators were used to combine keywords and Medical Subject Heading (MeSH) terms. Titles and abstracts were screened for relevance, and full texts of potentially pertinent articles were reviewed. Additional references were identified through manual searches of the cited literature. All included papers were evaluated by the authors for methodological quality, relevance, and contribution to the current understanding of the field. Findings were synthesized narratively to provide an integrated overview of existing evidence and emerging concepts.

For a cancer cell to be recognized by the immune system, cancer cell antigens have to be presented to T-cells through an antigen-presenting cell—cells that are able to provide a co-stimulatory signal. This interaction, alongside the major histocompatibility complex (MHC)-T-cell receptor (TCR) interaction, provides an activation signal to the T cells [2]. Activated T cells go on to start the immune response of T cells, along with an inhibitory system to ensure the inflammatory response does not go unchecked. This is achieved through immune checkpoints that provide an “off signal”. Among these, CTLA-4 was the first to be identified—a homolog of CD28 that binds B7-1 and B7-2 with a higher affinity [2]. Later, PD-1, which was initially identified through its involvement in negative selection in the thymus, was recognized as another checkpoint after the identification of its ligand PD-L1. PD-L1 was found on some cancer cells to induce T-cell apoptosis, protecting cancer cells from T cells [2,3]. Further understanding of the immune system identified other immune checkpoints, including lymphocyte activation gene 3 (LAG-3).

At the time of writing this review, there are two individual CTLA-4 inhibitors, seven individual PD-1 inhibitors, and four individual PD-L1 inhibitors with FDA approval for different cancer sites. A LAG-3 inhibitor, relatlimab, is currently approved in combination with the PD-1 blocker nivolumab for advanced melanoma [4]. Clinical trials are ongoing for other potential immune checkpoint targets such as TIM-3 and VISTA [2]. As such, the number of indications for immune checkpoint inhibitors has rapidly increased, with many overlaps between the different medications. This number increases further when different ICI are combined. Many of these indications are as first-line treatment across a range of malignancies, many times in combination with chemotherapy, targeted therapy and radiotherapy, greatly expanding their initial role as a “last resort” [5].

CAR T-cell therapy, another form of immunotherapy, involves engineered T lymphocytes that recognize malignant cells through the expression of chimeric antigen receptors directed against specific tumor-associated antigens [6]. These T cells are derived from patients and are activated ex vivo prior to CAR introduction [7]. These therapies can potentially be combined with ICI, which could help enhance their function through inhibition of the immunosuppressive microenvironment and reduction in CAR T-cell exhaustion from inhibitory checkpoint pathways [8].

As with most medical interventions, ICI are not without adverse events. In preclinical studies of ipilimumab, autoimmune phenomena such as prostatitis and depigmentation were noted in mice. During the early clinical studies, dermatitis, enterocolitis, hepatitis, hypophysitis, and uveitis were observed and later termed immune-related adverse events (irAEs) given their mechanism [9]. The incidence of all-grade irAEs is estimated to be 66% to 87%, higher in CTLA-4 inhibitors than in PD-1/PD-L1 inhibitors, and even higher when two ICI are combined [10]. The incidence of grade 3 or 4 irAEs is approximately 14–20% for PD-1/PD-L1 inhibitors and 29% for CTLA-4 inhibitors [10], increasing to 59% in ICI combination therapy [11].

A subtype of irAE related to the cardiovascular system, termed cardiovascular adverse events (CVAEs), was also noted to occur in patients receiving immunotherapy at a rate of approximately 1% [12]. These can include myocarditis, atherosclerotic disease, pericardial disease, arrhythmias, and heart failure, among others [13]. In this review, we aim to describe the mechanisms behind these CVAEs and describe their current clinical understanding.

## 2. Immune Checkpoint Inhibitor Myocarditis

### 2.1. Mechanisms

Advances in basic research have made it clear that the immune system plays a major role in cardiac homeostasis and repair after both ischemic and non-ischemic insults [14]. For example, Hayashi et al. showed in mouse models that an anti-PD-L1 antibody, combined with stressing mice using isoproterenol, led to increased myocardial inflammation [15]. The same stressor in PD-1−/− mice resulted in a prolonged inflammatory response, extended left ventricular (LV) dilation, and LV dysfunction compared to their wild-type counterparts. On a histopathological level, ICI-induced myocarditis presents as lymphocytic pericarditis, characterized by the infiltration of CD8+ and CD4+ T cells, along with CD68+ macrophages into the myocardium and conduction system [16,17]. However, the mechanisms through which this infiltration leads to injury remain unclear, though several theories have recently emerged. First, injury may occur through direct T-cell cytotoxic killing of myocytes, fibroblasts, endothelial cells, and mesothelial cells via molecular mimicry targeting an unidentified antigen [17], possibly α-myosin [18], along with increased levels of pro-inflammatory cytokines from activated immune cells and circulating cytokines [19,20]. Second, low levels of both PD-1 and PD-L1 are expressed on cardiomyocytes, raising the possibility of direct binding of certain ICI to these antigens, resulting in T-cell-mediated lysis of target cells [19,21]. Third, pre-existing autoantibodies may become unrestrained after blocking inhibitory pathways. Fourth, there may be increased systemic activation of T cells against antigens shared by both normal cardiac cells and malignant cells. Finally, although previously described in different forms of ICI-related irAEs but not specifically in cardiac cases, the theory of unmasking a subclinical microbial-induced inflammation present before ICI therapy has also been proposed [19].

### 2.2. Epidemiology

The largest meta-analysis investigating the incidence of ICI-associated cardiovascular adverse events comes from Nielsen et al. in 2024 [12]. It included 589 ICI clinical trials published up to 2023, encompassing 83,315 individual patients. Their analysis revealed the incidence of any-grade ICI-associated myocarditis to be 0.24% and of grade 3–4 myocarditis to be 0.18% [12]. This reported incidence of 0.24% lies within reported incidences in previous meta-analyses, which estimate the incidence of ICI-associated myocarditis to be between 0.12% and 3% [22,23,24,25,26,27]. Similar to other studies, the incidence of ICI-associated myocarditis was significantly higher in patients receiving dual ICI therapy [12].

Recently, there has been an increase in the reporting of ICI-associated myocarditis, attributed to improved recognition, the growing number of ICI, and expanding indications [28]. However, despite this increased reporting, the overall incidence of myocarditis in patients exposed to ICI has remained unchanged [12]. Utilizing the VigiBase database, Salem et al. conducted a pharmacovigilance study from the database’s inception until 2018, which included 122 patients. Their study showed a similar incidence rate of myocarditis at 0.31% for recipients of monotherapy ICI, rising to 1.31% for those receiving combination immunotherapy [29].

ICI-associated myocarditis usually develops within 12 weeks of treatment. It can occur as early as three days but can also develop more than 20 weeks later [12,29,30]. The median time of occurrence after the initiation of ICI was recently reported to be 23.5 days [12]. Symptoms suggestive of ICI-associated myocarditis include fatigue, chest pain, shortness of breath, orthopnea, lower-extremity edema, palpitations, light-headedness/dizziness, syncope, cardiogenic shock, or concomitant non-cardiac symptoms attributed to skeletal muscle injury, such as myalgias, diplopia, ptosis, and muscle weakness [30]. Of those, shortness of breath is the most reported (47%), followed by chest pain (14%) [12]. Other immune phenomena occurred concurrently in 65.5% of patients, including myositis (33.2%), followed by myasthenia gravis (31.8%) and increased liver enzymes/hepatitis (21.1%), along with other irAEs (34.5%) [12].

Data is lacking regarding risk factors for development of myocarditis after ICI. Braghiere et al., in their case control study, highlighted that absolute neutrophil count was associated with increased risk [31]. They did not find an association between classic cardiovascular risk factors such as diabetes and hyperlipidemia and risk of myocarditis [31], which has been replicated in other research [32]. Other reported risk factors include female gender, age over 75 years, combination of ICI treatment, and sleep apnea [32,33,34].

There is a significant mortality rate associated with ICI-associated myocarditis, reported in a meta-analysis to be 37.7% [12]. A more recent single center study reported a lower 2-year cardiovascular mortality of 6.6%, potentially reflecting timely treatment. However, it is also necessary to appreciate the small case number of 76 and single center design, alongside less severe cases. Yet, this mortality remained significantly higher than the non-myocarditis control group at 0.7% [31].

### 2.3. Diagnosis

The initial step in diagnosis is the early recognition of clinical signs and symptoms in the appropriate clinical context or early recognition through active monitoring of patients on ICI.

The definition of ICI-associated myocarditis was initially restricted to clinical trials, which used the Common Terminology Criteria for Adverse Events (CTCAEs) grading system to diagnose and stratify patients. After ICI-associated myocarditis was established as a concerning clinical entity, in 2019, Bonaca et al. proposed a definition that classified patients into definitive, probable, and possible ICI-associated myocarditis, a classification system also intended for research purposes [35]. More recently, the International Cardio-Oncology Society (IC-OS) released a consensus statement in 2022 that established the first diagnostic criteria intended for clinical practice [36] (Table 1).

In their definition, the current gold standard of diagnosis is histopathological diagnosis through an endomyocardial biopsy (EMB), showing the characteristic findings of ICI-associated myocarditis: immune infiltration (≥14 cells/mm^2^ or ≥7 CD3-positive T cells/mm^2^) and the presence of myocyte death [37]. Given the invasiveness and impracticality of performing EMB in many patients suspected for ICI-associated myocarditis, the 2022 consensus statement from the IC-OS offers clinical criteria that require an elevated troponin and cardiac magnetic resonance (CMR) imaging diagnostic for acute myocarditis (modified Lake Louise criteria), with clinical exclusion of acute coronary syndrome (ACS) and viral myocarditis, or a troponin elevation and meeting two out of five minor criteria shown in Table 1 [36]. In situations where these clinical criteria are not sufficient to establish the diagnosis, EMB should be considered [30].

Both troponin I and T can be used, but clinicians should be mindful that troponin T can be elevated in myositis without myocarditis. ECG findings include ventricular arrhythmias and/or new conduction system disease. Echocardiographic features of ICI-associated myocarditis include a decline in LV systolic function; however, 40% of patients have a preserved ejection fraction [12]. Other echocardiographic findings include presence of pericardial effusion, regional wall motion abnormalities, and decrease in the global longitudinal strain (GLS). A 2020 case-control study found that the mean GLS of patients who developed ICI-associated myocarditis decreased from a normal baseline (−20.5%) to −14.1% in both patients with reduced and preserved ejection fraction. The decrease in GLS was also associated with a 1.5- and 4.4-fold increase in major adverse cardiovascular events (MACE) among patients with a reduced EF and preserved EF, respectively [38]. Another more recent study found that magnitude of GLS reduction correlated with the risk of MACE [39].

CMR is the gold standard noninvasive diagnostic tool for ICI-associated myocarditis. The diagnosis currently utilizes the modified Lake Louise criteria for cardiac inflammation [30]. CMR findings can vary from a lack of features to diffuse inflammation [40]. In 2017, Escudier et al. published the CMR findings in a series of 30 patients from two centers. They reported late gadolinium enhancement (LGE) in 23% and myocardial edema on T2-STIR sequences in 33% [41]. The largest international multicenter cohort evaluating CMR was published in 2020 by Zhang et al., which included 103 patients from 23 centers. They reported LGE in 42% of patients with ICI-associated myocarditis, and 28% had an elevated T2-STIR signal [42]. Similar to the study by Mahmood et al., the distribution of LGE was also variable [43]. This study raised a few important points. First, presence of LGE did not correlate with a composite outcome of cardiovascular death, cardiogenic shock, cardiac arrest, and complete heart block, and could not predict severity. Second, it was observed that the more delayed the CMR after presentation, the more likely LGE was detected. For example, when CMR was done on day four of presentation, the detection of LGE increased from 21.6% to 72.0%. However, as will be discussed later, early diagnosis and treatment are associated with improved outcomes [42,44]. As such, delaying the CMR or relying solely on CMR to exclude ICI-associated myocarditis is not recommended, particularly in early presentations. Finally, Thavendiranathan et al. found that when applying the modified Lake Louise Criteria with T1 and T2 mapping, 95% met the nonischemic myocardial injury criteria and 53% met the myocardial edema criteria. Native T1 values had excellent discriminatory value for subsequent MACE, with an area under the curve of 0.91. Native T1 values were also independently associated with subsequent MACE [45].

Despite the invasive nature of EMB, having a lower threshold to perform an EMB is recommended in cases where clinical suspicion remains high, or directly performing an EMB in severe cases where early timely diagnosis is paramount, as histopathological diagnosis remains the gold standard [42]. The histopathologic findings are not clearly explored in the literature in the context of ICI myocarditis. The Dallas criteria represent the traditional histopathological standard for diagnosing myocarditis, requiring evidence of cardiomyocyte necrosis or degeneration in conjunction with an inflammatory infiltrate [46]. In contrast, Palaskas et al., in a retrospective analysis of 28 patients undergoing EMB, proposed a classification scheme that captures a spectrum of disease severity, ranging from low-grade inflammatory infiltrates without myocyte loss to high-grade inflammation with myocyte loss [16]. This approach may identify patients with early-stage myocarditis who would not meet Dallas criteria, potentially increasing diagnostic sensitivity. Nevertheless, the prognostic and therapeutic implications of detecting early inflammatory changes remain uncertain, underscoring the need for further studies to determine the clinical utility of graded histopathological systems.

### 2.4. Treatment

There is a lack of randomized controlled trials and high-quality evidence to guide the treatment of ICI-associated myocarditis, and recommendations are based on experience derived from other irAEs and the treatment of cardiac transplant rejection [12].

In 2022, the European Society of Cardiology released guidelines on cardio-oncology, which recommend temporarily holding the ICI until diagnosis is confirmed or refuted and starting 500–1000 mg/day IV methylprednisolone as early as possible, ideally within 24 h, and continuing for three days [30]. The recommendation regarding early and high-dose corticosteroids use is based on a 2020 international multicenter retrospective study by Zhang et al. on 126 patients, which categorized patients based on corticosteroid dose (low, intermediate, high) and timing (≤24 h, 24–72 h, and >72 h). The study showed that patients who received high-dose corticosteroids and those who received corticosteroids early had a significantly lower incidence of MACE, a composite outcome that included cardiovascular death, cardiac arrest, cardiogenic shock, and hemodynamically significant complete heart block requiring a pacemaker [44].

After three days of high-dose corticosteroids, patients with a troponin reduction of >50% from peak and stable clinical condition (no new-onset heart failure, complete heart block, or ventricular arrhythmias) are classified as uncomplicated ICI-associated myocarditis. These patients can then be switched to oral prednisone 1 mg/kg/day (max 80 mg/day) with a weekly taper of 10 mg per week, along with weekly monitoring of troponin and ECG. Patients who, after three days, have a troponin reduction of <50% or a rising troponin, hemodynamic instability, new-onset heart failure, cardiogenic shock, complete heart block, or ventricular arrhythmias are classified as complicated ICI-associated myocarditis. In these patients, IV methylprednisolone 1000 mg/day is continued, and second-line agents are initiated, such as mycophenolate mofetil or tocilizumab (8 mg/kg), anti-thymocyte globulin, alemtuzumab, or abatacept. Other agents investigated include intravenous immunoglobulin (IVIG), plasma exchange, rituximab, and tacrolimus [30,47]. There is no clear evidence comparing these agents, and studies are ongoing. Of note, infliximab was shown as having an increased risk of worsening existing heart failure, and therefore, the 2022 guidelines advised its avoidance in heart failure cases until further data emerge [47]. These recommendations are consistent with other expert society guidelines, such as those from the National Comprehensive Cancer Network [48], the Society for Immunotherapy of Cancer [49], and the European Society for Medical Oncology [50].

Another approach is taken by the American Society of Clinical Oncology (ASCO), which, in its 2021 guidelines, recommends starting with high-dose corticosteroids (1–2 mg/kg/day of prednisone, oral or IV) and escalating the dose to IV methylprednisolone 1000 mg/day only in patients without an immediate response, with the addition of mycophenolate, infliximab, or anti-thymocyte globulin. For life-threatening cases, they recommend considering the addition of abatacept or alemtuzumab [13].

The 2025 ESC guidelines for myocarditis and pericarditis similarly advise rapid triage and steroid initiation [51], However, they suggest initiation of second line therapy after 24 to 48 h of no response to steroid treatment, a class IIa recommendation, and in fulminant or severe cases, as class IIb. Their guidance further includes third line therapy options of infliximab, adalimumab or rituximab. In addition, they more clearly separate steroid dosing based on disease severity, with weight-based dosing for severe disease. More recently, the 2026 IC-OS position statement goes further and recommends that for patients with severe or fulminant disease to start directly with combination therapy of high dose steroids and another immunosuppressant: abatacept with or without a JAK inhibitor, anti-thymocyte globulin, IVIG or plasma exchange. This recommendation was also extended to patients presenting with tripleM syndrome (myositis, myocarditis, and myasthenia gravis) [52]. Additionally, the management of troponin elevation of uncertain significance, smoldering myocarditis, nonsevere myocarditis was also discussed. Specifically, troponin elevation of uncertain significance should not prompt discontinuation of ICI therapy or initiation of immunosuppression. In the situation of smoldering myocarditis (meeting diagnostic criteria of ICI myocarditis but remaining asymptomatic with minimal abnormities), ICI should initially be held but may be re-started after multidisciplinary review if it remains the best option for cancer treatment. Serial monitoring of cardiac biomarkers, ECG, and signs and symptoms are warranted. Nonsevere myocarditis can be treated as severe myocarditis initially, but faster tapers of corticosteroids can be considered [52].

Given the mechanism of action of ICI, abatacept, a CTLA4–Ig fusion protein that acts as a decoy receptor for CD80/CD86 expressed on antigen-presenting cells, has recently emerged as a potential treatment, as it can lead to global T-cell anergy. In a 2024 case series by Salem et al. that compared standard steroid therapy with the addition of ruxolitinib and abatacept to steroids in 40 patients, a statistically significant decrease in ICI-associated myocarditis mortality was observed, from 6/10 (60%) to 1/30 (3%) [53]. Other case reports have shown success using a similar approach in steroid-refractory patients [54]. A phase II dose-finding clinical trial is currently ongoing to determine the appropriate dosing for abatacept (AbataCept for the Treatment of Immune-cHeckpoint Inhibitors-Induced mYocarditiS [ACHLYS], ID NCT05195645) [55]. In addition, the ATRIUM (AbatacepT foR ImmUne-checkpoint-inhibitor-associated Myocarditis) trial, a phase III randomized, double-blind, placebo-controlled study is ongoing to compare abatacept with placebo for reduction of MACE in hospitalized ICI myocarditis patients (ID NCT05335928) [56]. The addition of ruxolitinib, a JAK1/JAK2 inhibitor frequently used in graft-versus-host disease, is due to its synergy with abatacept and its rapid onset of action, as abatacept has a slow onset of clinical efficacy [53].

### 2.5. Monitoring

Guidelines recommend that all patients planned to be treated with ICI should undergo a baseline clinical cardiovascular assessment, including an evaluation of symptoms, physical examination, cardiovascular risk factors, ECG, natriuretic peptides, and cardiac troponin levels. Patients at an elevated risk for ICI myocarditis may be considered for a baseline echocardiogram. Troponin and ECG surveillance before second, third and fourth doses of ICI is recommended only by the ESC 2022 cardio-oncology guidelines as a class IIa indication [30]. This approach is still controversial given the low incidence of ICI myocarditis and uncertain cost-effectiveness of this approach. Additionally, clinicians should avoid discontinuing ICI prematurely if these screening tests become positive because many diagnoses other than ICI myocarditis can incur similar findings and a careful decision plan should be made by a multidisciplinary team to optimize cancer and cardiovascular outcomes. Patients are determined to be at a higher risk of ICI myocarditis include those on dual ICI treatment, ICI combined with other cardiotoxic agents, patients experiencing other non-cardiac irAEs, patients with prior cardiac toxicities related to cancer therapy, and patients with baseline cardiovascular disease [30]. Using ECG and troponin surveillance in a selected high-risk population is probably more reasonable.

A surveillance program was initiated at the Stanford Cancer Institute, incorporating active monitoring of cardiac enzymes for patients on ICI therapy, enrolling 214 patients. This program successfully identified three patients who were detected early and treated appropriately, leading to favorable outcomes [57]. This adds to the body of evidence supporting the importance of early recognition and treatment.

Long-term cardiovascular outcomes after the development of ICI myocarditis are worsened compared to ICI users who do not experience myocarditis. In a study by Braghieri et al., which evaluated 76 patients with myocarditis and compared them to those without myocarditis, they found a lower two-year freedom from cardiovascular composite outcome of new-onset heart failure, atrial fibrillation, cerebrovascular accidents or myocardial infarction (67% vs. 86.8%) and lower two-year freedom from cardiovascular death (93.4% vs. 99.3%) in the myocarditis group [31]. Mortality is also high in patients who develop pericardial disease, with a reported incidence of 21% [29]. Among 424 patients receiving ICI therapy, those who subsequently developed new cardiovascular disease had a significantly higher mortality rate compared to those who did not (66.1% vs. 41.4%; OR 2.77, 95% CI 1.55–4.95) [58]. These findings suggest a substantial decline in survival among patients who experience cardiovascular complications from ICI, likely due to the interplay between cardiac dysfunction and underlying malignancy.

Most patients who experience severe ICI myocarditis are generally advised against rechallenging with ICI because of the high risk of recurrence. In cases where patients have recovered from myocarditis and have maintained preserved cardiac function, there may be a possibility for rechallenge, particularly if the myocardial injury is of a lower grade as determined by EMB. However, this decision must be made on a highly individual basis, weighing the potential benefits of continuing cancer treatment against the risks of exacerbating cardiac pathology. The 2025 ESC myocarditis and pericarditis guidelines reiterate the importance of a multidisciplinary approach to rechallenge, with consideration of the aforementioned elements [51].

## 3. Accelerated Atherosclerotic Cardiovascular Disease

Atherosclerotic cardiovascular disease (ASCVD) has been reported in 4.9% of patients using ICI, compared to 1.4% in matched non-ICI users [59]. In this 2024 study by Gong et al., patients with non-small cell lung cancer with and without ICI use were matched (*n* = 446), and there was no significant difference in baseline characteristics. When assessing coronary artery calcium volume, at 12 months there was more significant progression in the ICI group (30.4% relative volume progression vs. 19.7%; *p* = 0.004). A single-center cohort study by Drobni et al. in 2020 with 2842 matched patients also demonstrated that patients receiving ICI exhibited a significantly elevated risk of ASCVD events, including myocardial infarction and the need for coronary revascularization (HR 3.3; 95% CI 2.0–5.5) [60]. Imaging revealed increased plaque volume during the study period, with an escalation in the rate of atherosclerotic plaque progression following initiation of ICI, from 2.1% per year to 6.7% per year.

Immune checkpoints have been identified on cellular components within atherosclerotic lesions, and their inhibition by ICI can promote an inflammatory response and plaque progression [61,62]. Autopsy findings in patients treated with ICI revealed an increased ratio of CD3+ T cells to CD68+ macrophages within atherosclerotic plaques, highlighting a potential mechanistic link wherein ICI induced alterations in plaque composition predispose the patient to accelerated disease progression [63]. Under physiological conditions, immune checkpoints contribute to limiting atherosclerotic lesion formation and attenuating T-cell-mediated inflammation. Consequently, their inhibition by ICI may disrupt this mechanism, potentially accelerating the progression of atherosclerosis [64]. Further research is required to more clearly delineate these pathophysiologic mechanisms.

## 4. Heart Failure

While myocarditis is the most commonly reported cardiac irAE, heart failure can occur following ICI treatment even in the absence of myocarditis, which could be due to Takotsubo syndrome, pericardial disease, arrhythmias, or vascular complications. Noninflammatory heart failure, or non-inflammatory left ventricular dysfunction (NILVD), can occur as a complication of ICI therapy, and it is characterized by a reduced LVEF without evidence of active inflammation on cardiac imaging or elevated cardiac troponin levels. In contrast to ICI myocarditis, most patients with NILVD had normal troponin, no active inflammation on CMR and few had other irAEs. While the exact mechanisms are not fully understood, ICI-related cardiotoxicity may involve direct myocardial damage, endothelial dysfunction, and accelerated atherosclerosis. It remains less frequently reported and explored in the literature compared to other cardiotoxicity. The incidence of heart failure in ICI users was reported as 0.72% in an analysis of the VigiBase database by Salem et al., which was comparable to the overall incidence within the database of 0.87% [29]. Elsewhere, reported rates vary from 1.49% in a retrospective review of 538 patients [65] to 3.49% in a review of patients from the IBM MarketScan research databases, which included 16,574 receiving ICI [66]. In that database study, anti-CTLA-4 monotherapy or combination therapy had a 1.5–2 folds higher risk of heart failure compared to anti-PD-1 therapy. Andres et al. reported that NILVD occurred in 15 patients out of the 89 that experienced cardiovascular adverse events from ICI therapy, from a cohort of 2647 patients treated with ICI in the absence of myocarditis, ischemia, infarction, or other acute causes [67]. Compared to ICI myocarditis, the main differences in management are that immunosuppression therapy is not indicated and ICI treatment can be restarted once the heart failure syndrome has been stabilized [68].

## 5. Cardiomyopathy

Dilated cardiomyopathy has been reported in association with ICI use, as demonstrated by a case report of an 88-year-old woman receiving nivolumab for metastatic melanoma and with no cardiac history [69]. There has been a report on diastolic dysfunction with a restrictive pattern following ipilimumab treatment for metastatic melanoma in an 80-year-old man [70]. However, data on ICI-associated cardiomyopathy remain limited, and its occurrence may be linked to myocarditis [71]. Notably, mice deficient in PD-1 have been shown to develop dilated cardiomyopathy, supporting the hypothesis that autoimmune T cells activated by ICI may target cardiac myosin, predisposing individuals to this condition [72]. Autoantibodies to cardiac troponin I in PD-1 deficient mice have also been linked to dilated cardiomyopathy [73].

## 6. Stress-Induced Cardiomyopathy

Stress-induced cardiomyopathy, also known as Takotsubo cardiomyopathy, is a rare complication associated with ICI therapy. It has been documented in case studies [74,75,76], including a report of a 76-year-old man with urothelial carcinoma who went on to develop Takotsubo cardiomyopathy after receiving atezolizumab [75]. The diagnosis of the condition can be challenging, given the need to exclude ACS and myocarditis, and it is not well reported in the literature. Although the mechanism behind this complication is not established, several theories have been proposed. These include a catecholamine surge leading to cardiac stunning, direct ICI-induced injury to myocardial or coronary tissue, and T-lymphocyte-mediated inflammatory changes [76,77].

## 7. Arrhythmias

Multiple arrhythmias have been associated with ICI therapy, including ventricular tachycardia, atrial fibrillation, and AV block [78]. A subset of these arrhythmic events occurs in the context of myocarditis. However, myocarditis may not be the only explanation for the increased arrhythmic risk with ICI, and other pathophysiologic mechanisms may be considered. A retrospective study from Heemelaar et al. included 7441 patients using ICI, when evaluating a composite of any arrhythmia compared to a control population, they found a higher arrhythmic risk over a follow-up period of 36 months (HR 1.68, 95% CI 1.39–2.04) [79]. This was in a population without myocarditis, indicating a separate mechanistic pathway of ICI-induced arrhythmia. Analysis of the FDA Adverse Event Reporting System between 2011 and 2021 showed 1945 cases of ICI-related arrhythmias, of which 26% died [78]. Both anti-PD-1 and PD-L1 agents were associated with arrhythmic events, whereas anti-CTLA-4 or combination immunotherapy did not. Proposed theories include increased production of inflammatory cytokines such as IL-6, which may predispose to arrhythmias [79,80], as well as ICI-induced cardiac fibrosis [81]. It is also possible that PD-1 itself may have direct impact on the development of arrhythmias, particularly atrial fibrillation. More research is needed to better understand the pathophysiology of ICI mediated arrhythmias.

## 8. Pericardial Disease

Data from the FDA Adverse Event Reporting System reveal that pericardial disease, which encompasses pathology ranging from pericardial disease to pericardial effusion, was reported in 705 cases among patients receiving ICI between 2011 and 2020 [82]. This was predominantly an early onset complication, with the median time from initiation of treatment to incidence being 38 days. However, delayed pericardial disease has also been reported, with a case report reporting nivolumab-induced pericarditis 18 months after initiation [83]. A 2024 systematic review from Mudra et al. evaluated 31 studies, which presented 38 patients who developed ICI-related pericardial disease [84]. Data from this study and the FDA are consistent in indicating a higher prevalence of pericardial disease in males and in patients with lung cancer.

A larger 2025 retrospective study utilizing a large dataset from a global network included 88,928 patients treated with ICI. It showed an incidence of pericarditis at 0.22%, tamponade at 0.47%, and a higher incidence of pericardial effusion at 4.71% [85]. One-year mortality of patients experiencing pericardial disease was significantly higher compared to matched controls (HR 1.46, 95% CI: 1.37–1.56). These findings are further supported by a single center study by Gong et al., which included 2842 patients treated with ICI matched to non-ICI users [86]. They demonstrated an incidence of pericardial events of 1.48% in ICI users, with a notably increased risk compared to non-ICI users (HR 4.37, 95% CI 2.09–9.14). A smaller matched study also aligns with these findings, showing an increased incidence of pericarditis (HR 1.51, 95% CI: 1.03–2.22) [87].

Multiple mechanisms underlying pericardial disease have been proposed, though they remain unclear. One potential cause is a loss of self-tolerance of T cells, leading to immune-mediated attack of tissue and subsequent inflammatory pathology [88]. In addition, certain patients, such as those with lung cancer, may undergo radiotherapy, predisposing them to higher risk of pericardial effusion and exposing pericardial antigens that may then be recognized by T lymphocytes [84].

## 9. CAR T-Cell Cardiotoxicity

CAR T-cell therapy has been associated with cardiotoxicity. The spectrum of cardiotoxicity includes new left ventricular dysfunction, heart failure, arrhythmias, conduction abnormalities, pericardial effusion, takotsubo cardiomyopathy, and cardiac arrest [68]. In a prospective study of 27 patients, the incidence of cancer therapy associated cardiac dysfunction (CTRCD) at 7 days was 59.3% [89]. This included a combination of cardiac biomarker abnormality or reduction in GLS or LVEF. In a retrospective review of 75 patients by Patel et al., 12% experienced cardiac events within 1 year of treatment [90]. A meta-analysis of 20 studies involving 4789 patients revealed a 19.68% incidence rate of cardiovascular events, with arrhythmias (7.70%), heart failure (5.73%), and reduced LVEF (3.86%) being the most prevalent. Troponin elevation was observed in 23.61% of patients, while NT-Pro-BNP elevation was observed in 9.4% [91].

### 9.1. Mechanisms

Cytokine release syndrome (CRS) is a predominant complication of CAR T-cell therapy and is likely secondary to release of cytokines from these T cells due to rapid activation and proliferation, leading to greater immune cell recruitment and further cytokine secretion [92]. This cytokine increase may also be secondary to tumor-cell lysis, and the resultant increase in both cytokine and chemokines is thought to contribute to cardiotoxicity [93]. IL-6, a key driver of CRS, may impair cardiac function through multiple pathways, including oxidative stress, cardiomyocyte apoptosis, and depressed contractility [93,94]. Other proposed contributing elements to cardiac dysfunction include capillary leak through upregulation of angiopoietin-2, and cardiac hypertrophy and heart failure with prolonged elevations of IL-6 [94,95]. In the Camilli et al. study, 88.8% of the patients that had experienced CTRCD had CRS, suggesting a potential contributing factor and a greater relation with more severe CRS. In addition to the on-target immune activation responsible for CRS, CAR T-cell therapy may theoretically cause direct cardiovascular injury through off-tumor activity. This may occur through on-target recognition of an antigen shared by malignant and cardiovascular tissues or through off-target cross-reactivity with an unrelated cardiac antigen. These mechanisms could result in direct T-cell-mediated myocardial injury, but this mechanism remains under explored in the literature [95].

### 9.2. Risk Factors

Few studies explore risk factors for CAR T-cell therapy cardiotoxicity. History of atrial arrhythmia increased the risk of future arrhythmia, lower baseline GLS and higher E/e′ was associated with increased cardiac events [90], and baseline creatinine was associated with MACE risk. However, it should be noted that features of CRS include hypotension and tachycardia, which may further complicate the picture of cardiotoxicity identification.

### 9.3. Monitoring

Similar to those receiving ICI therapy, patients who require CAR T-cell therapy must undergo baseline ECG, natriuretic peptide, and troponin checks. A comprehensive transthoracic echocardiogram should be considered in all patients receiving CAR T-cell therapy, and those with pre-existing cardiovascular disease or prior cardiotoxic treatment such as anthracyclines are recommended to have a baseline echocardiogram [30,68]. After therapy is initiated, focus is on early recognition of CRS and cardiovascular complications. This includes regular assessment of hemodynamic monitoring and signs of heart failure or arrhythmias. Repeat ECG, natriuretic peptide, and troponin testing is advised, and for those at high risk for cardiovascular events, consideration can be made for repeat echocardiogram [68]. Heart Failure Association (HFA) of the ESC and the ESC council of cardio-oncology suggests repeating ECG and troponin 2–5 days and 2–3 weeks post infusion to identify cardiac abnormalities and repeating echocardiogram 2–3 weeks post-infusion, especially in patients with ≥grade 2 CRS [68]. Inflammatory markers such as C-reactive protein and ferritin may help assess the systemic inflammatory response but are not specific for cardiac injury [95]. CMR imaging may be considered when myocardial dysfunction or biomarker elevation persists and the diagnosis remains uncertain to assess inflammation or fibrosis [68].

### 9.4. Limitations

The current studies evaluating CAR T-cell therapy have relatively small sample sizes, and given that this is a new therapy modality, further data is needed to establish clear guidelines regarding risk assessment and monitoring for cardiotoxicity. This is particularly important with consideration made for possible combination with ICI treatment and expanding indications for CAR T-cell therapy, which may lead to increased cardiotoxic effects.

## 10. Emerging Risk-Stratification Strategies

New tools are emerging to better predict risk of cardiotoxicity with ICI and CAR T-cell therapy. In a retrospective cohort of 375 patients, a 0–4 multimodal score incorporating pretreatment cardiac troponin T, N-terminal pro-B-type natriuretic peptide, high-sensitivity C-reactive protein, and coronary artery calcium score demonstrated a graded association with subsequent cardiovascular toxicity, with the highest score associated with increased ICI cardiotoxicity [96]. A score of 4, as compared to 0–1, was associated with a 7.29-fold increase in risk of cardiomyopathy and an 8.83-fold increase in heart failure or myocarditis. Li et al. demonstrated a different approach, with their utility of XGBoost [97]. This machine learning model was evaluated using 491 patients to predict ICI cardiotoxicity within 30 days, defined by cardiac biomarker and ECG abnormality. The area under the curve (AUC) was 0.83, and troponin T emerged as the most influential predictor. More recently, a multicenter lung cancer-specific nomogram incorporating baseline troponin, natriuretic peptide, ECG, and TTE abnormalities demonstrated good internal and external discrimination for severe ICI-associated myocarditis [98]. AUC in the external validation set was 0.95, but assessment in other cancer populations remains necessary. In patients who have developed myocarditis, a prognostic score was developed by Power et al. to predict poor outcomes [99]. This risk score incorporated active thymoma, cardiomuscular symptoms, low QRS voltage, left ventricular ejection fraction under 50%, and the magnitude of troponin elevation, and they found a stepwise increase in 30-day major cardiomyotoxic events from 4% at score 0 to 81% at score ≥ 4.

Specific to CAR T-cell therapy is the CART-7 score, which is designed to predict cardiovascular adverse events [100]. This proposed system incorporated seven elements: coronary artery disease, hyperlipidemia, diabetes, hypertension, atrial fibrillation, heart failure, and smoking. It was derived from meta-analysis of 12 studies including 1354 patients with hematologic malignancies, and results indicate increasing risk of events with high scores. As with ICI therapy, there have been multiple computational models studied for therapy-related adverse events. A 2026 meta-analysis identified 26 such models, but all of which were judged to have a high risk of bias. These were predominantly to predict risk of CRS, but given the relationship between CRS and cardiotoxicity, such predictive models may potentially be applicable to cardiotoxicity. There is a lack of data currently available to explore this [101].

These upcoming prognostic tools promise improved ability to identify high-risk patients and thus potentially target preventive strategies. Through identification of those at increased risk of poor outcomes after development of toxicity, they may also help direct treatment strategies. However, the current tools should complement, rather than replace, guideline-based surveillance.

## 11. Future Directions

Evidence supports a spectrum of cardiotoxicity associated with both ICI and CAR T-cell therapies; however, most data are derived from retrospective analyses. While these studies provide high-level epidemiologic data, there remains a need for prospective trials. One such area is the evaluation of the efficacy and safety of ICI myocarditis treatment options. This necessitates a higher quality of data, such as randomized controlled trials, to aid with identification of optimal treatments of complications.

Risk stratification is another area that requires further development. Although several risk models have recently been proposed for ICI and CAR T-cell therapy associated cardiovascular toxicity, their clinical implementation remains limited by heterogeneous outcome definitions, small event numbers, and insufficient prospective external validation. Further research should determine whether these models can reliably guide individualized cost-effective surveillance strategies and improve clinical outcomes.

ICI therapy, and to a greater extent CAR T-cell therapy, are relatively new treatment modalities. Although their use is becoming increasingly common, long-term outcome data are limited. Consequently, the prognosis and true long-term risk of cardiotoxicity associated with these modalities are not yet well defined. Future research will aim to provide a more comprehensive evaluation of these complications.

Additionally, the advent of artificial intelligence and machine learning offers new opportunities to predict, detect, diagnose, and manage cancer immunotherapy cardiotoxicity through complex patten recognition, multimodal electronic health record integration, automated imaging, and biomarker and multi-omics triangulation. Although the field is currently significantly limited by retrospective designs and lack of external validation, and effective clinical implementation, it will be further investigated and explored for its clinical utilization. Responsible research and robust validation will be necessary to transition these innovations into everyday clinical practice.

## Figures and Tables

**Table 1:** Diagnostic criteria for ICI-associated myocarditis (adapted from the 2022 International Cardio-Oncology Society (IC-OS) consensus statement).

Diagnosis Type	Criteria
Pathohistological diagnosis (endomyocardial biopsy)	Multifocal inflammatory cell infiltrates with overt cardiomyocyte necrosis or loss on light microscopy.
Clinical diagnosis	Elevated cardiac troponin (new rise or significant change from baseline) plus either one major or two minor criteria, after excluding acute coronary syndrome and infectious myocarditis.
	Major Criterion: Cardiac magnetic resonance (CMR) diagnostic for acute myocarditis (meeting modified Lake Louise criteria: T2-based myocardial edema and non-ischemic LGE) Minor Criteria: Compatible clinical syndrome (fatigue, myalgia, chest pain, dyspnea, orthopnea, edema, palpitations, syncope, muscle weakness, diplopia, ptosis, or cardiogenic shock) Ventricular arrhythmia or new conduction system disease (e.g., AV block, bundle branch block) New decline in LV systolic function or regional wall-motion abnormality in a non-Takotsubo pattern Other immune-related adverse events (myositis, myopathy, myasthenia gravis) CMR findings suggestive but not fully diagnostic of myocarditis

Adapted from Herrmann, J.; Lenihan, D.; Armenian, S.; Barac, A.; Blaes, A.; Cardinale, D.; Carver, J.; Dent, S.; Ky, B.; Lyon, A.R.; et al. Defining cardiovascular toxicities of cancer therapies: An International Cardio-Oncology Society consensus statement. Eur. Heart J. 2022, 43, 280–299. https://doi.org/10.1093/eurheartj/ehac244 [36].

## Data Availability

No new data were created or analyzed in this study. Data sharing is not applicable to this article.
